# Risk of hernia formation after radical prostatectomy: a comparison between open and robot-assisted laparoscopic radical prostatectomy within the prospectively controlled LAPPRO trial

**DOI:** 10.1007/s10029-020-02178-7

**Published:** 2020-04-11

**Authors:** H. Nilsson, J. Stranne, J. Hugosson, C. Wessman, G. Steineck, A. Bjartell, S. Carlsson, T. Thorsteinsdottir, S. I. Tyritzis, A. Lantz, P. Wiklund, E. Haglind

**Affiliations:** 1 Department of Surgery, Institute of Clinical Sciences, SSORG, Scandinavian Surgical Outcomes Research Group, Sahlgrenska Academy, University of Gothenburg, Gothenburg, Sweden; 2 Department of Surgery, Region Västra Götaland, Sahlgrenska University Hospital, Gothenburg, Sweden; 3 Department of Urology, Institute of Clinical Science, Sahlgrenska Academy, University of Gothenburg, Gothenburg, Sweden; 4 Department of Urology, Region Västra Götaland, Sahlgrenska University Hospital, Gothenburg, Sweden; 5 Sweden and Health Metrics Unit, The Sahlgrenska Academy, University of Gothenburg, Gothenburg, Sweden; 6 Division of Clinical Cancer Epidemiology Institute of Clinical Sciences, Sahlgrenska Academy At the University of Gothenburg, Gothenburg, Sweden; 7 Department of Oncology and Pathology, Division of Clinical Cancer Epidemiology, Karolinska Institutet, Gothenburg, Sweden; 8 Department of Urology, Skåne University Hospital, Lund University, Gothenburg, Sweden; 9 Faculty of Nursing, Research Institute in Emergency Care, Landspitali University Hospital, University of Iceland, Reykjavik, Iceland; 10 Department of Molecular Medicine and Surgery, Section of Urology, Karolinska Institutet, Gothenburg, Sweden; 11 Department of Surgery, Sahlgrenska University Hospital/Östra, Östra Sjukhuset, 416 85 Göteborg, Sweden

**Keywords:** Inguinal hernia, Incisional hernia, Radical prostatectomy

## Abstract

**Purpose:**

In addition to incisional hernia, inguinal hernia is a recognized complication to radical retropubic prostatectomy. To compare the risk of developing inguinal and incisional hernias after open radical prostatectomy compared to robot-assisted laparoscopic prostatectomy.

**Method:**

Patients planned for prostatectomy were enrolled in the prospective, controlled LAPPRO trial between September 2008 and November 2011 at 14 hospitals in Sweden. Information regarding patient characteristics, operative techniques and occurrence of postoperative inguinal and incisional hernia were retrieved using six clinical record forms and four validated questionnaires.

**Results:**

3447 patients operated with radical prostatectomy were analyzed. Within 24 months, 262 patients developed an inguinal hernia, 189 (7.3%) after robot-assisted laparoscopic prostatectomy and 73 (8.4%) after open radical prostatectomy. The relative risk of having an inguinal hernia after robot-assisted laparoscopic prostatectomy was 18% lower compared to open radical retropubic prostatectomy, a non-significant difference. Risk factors for developing an inguinal hernia after prostatectomy were increased age, low BMI and previous hernia repair. The incidence of incisional hernia was low regardless of surgical technique. Limitations are the non-randomised setting.

**Conclusions:**

We found no difference in incidence of inguinal hernia after open retropubic and robot-assisted laparoscopic radical prostatectomy. The low incidence of incisional hernia after both procedures did not allow for statistical analysis. Risk factors for developing an inguinal hernia after prostatectomy were increased age and BMI.

**Electronic supplementary material:**

The online version of this article (10.1007/s10029-020-02178-7) contains supplementary material, which is available to authorized users.

## Introduction

Radical prostatectomy has been associated with an increased risk of inguinal hernia formation postoperatively for more than two decades. Regan et al. first described a 12% incidence of inguinal hernia within 6 months of open retropubic radical prostatectomy (RRP) [1] that was later confirmed by several independent reports [2–5]. The cause, however, remains unknown. It has been suggested that it is the lower midline incision that induces the inguinal hernia formation and that all surgery performed by a lower midline incision could cause this complication [6]. Today a majority of prostate cancer operations are performed using robot-assisted laparoscopic technique (RALP) and hence several authors have addressed the question whether the risk of postoperative inguinal hernia is increased to the same extent after robot-assisted laparoscopic radical prostatectomy, reporting diverging results [5, 7–10].

Incisional hernia is a well-known complication to all types of abdominal surgery [11, 12]. Reports on incisional hernia rates after prostate cancer surgery have suggested an increased risk of developing an incisional hernia after RALP as compared to RRP, despite the longer incision of the latter [8, 10, 11, 13]. Inguinal and incisional hernia may infrequently cause severe morbidity, and even mortality [14, 15]. Radical prostatectomy has become one of the most commonly performed surgical procedures, and the patient focused interest to reduce complications such as incisional and inguinal hernia formation postoperatively can also be regarded as of socioeconomic interest, to reduce societal resource consumption.

The aim of this report was to compare the cumulative rate of inguinal and incisional hernia, respectively, within 24 months after RRP and RALP in the setting of a prospective, controlled trial of the two techniques, LAPPRO.

## Patients and methods

Patients with localized prostate cancer operated by radical prostatectomy at 14 centers in Sweden were prospectively included in the LAPPRO trial (trial registration number: ISRCTN06393679) between September 2008 and November 2011. Seven centers performed RRP and seven other centers performed RALP with the primary endpoint of the LAPPRO trial being urinary incontinence at 12 months [16]. Data regarding patient characteristics, operative findings and postoperative wellbeing was retrieved using six clinical record forms and four questionnaires. Exclusion criteria were age above 75 years, PSA above 20 ng/ml, tumor stage above cT3 as well as inability to understand Swedish. Ethical approval was obtained from the Regional Ethical review board in Gothenburg No 277-07. The trial protocol can be found at www.ssorg.net.

### Demography and patient characteristics

Information regarding patient characteristics were retrieved from the preoperative questionnaire. Information included in analyses were age, body mass index (BMI), diabetes (yes/no), cardiovascular disease (yes/no), pulmonary disease (yes/no), smoking/non-smoking, tumor stage, physical activity prior to surgery and previous hernia repair. Cardiovascular disease (yes/no) is a derived variable consisting of hypertension (yes/no), cardiac failure (yes/no), and myocardial infarction (yes/no).

Physical activity prior to surgery was derived from the following question: how often have you been physical active for more than 30 min during the last month? (1) never, (2) 1–2/week, (3) 3–4 times/week and (4) 5–7/week). The answers were dichotomized into rarely (answer 1–2) and often (category 3–4).

### Outcome variables

Patients answered validated questionnaires at baseline and at 3, 12 and 24 months after surgery including specific questions regarding inguinal hernia [16] and six clinical record forms were filled out by hospital staff, including information about any type of additional surgery within 24 months. Patients were examined by a urologist before surgery and followed up at 3, 12 and 24 months after prostatectomy where any groin hernia were noted in clinical record forms. All data were collected in a trial database. Information was retrieved regarding prevalent inguinal hernia at the time of radical prostatectomy, previous inguinal hernia repair and postoperative development of inguinal hernia as well as any inguinal hernia repair within 24 months. Likewise, information regarding incisional hernia was retrieved through the patient questionnaires and from clinical record forms at baseline, perioperatively and 3, 12 and 24 months after prostatectomy.

Inguinal hernia was defined as any clinical appearance of or operation for an inguinal hernia after the index prostatectomy reported in clinical record forms or patient questionnaire at 3, 12 or 24 months after surgery. Correspondingly, incisional hernia was defined as any clinical appearance of or operation for incisional hernia after the index prostatectomy reported in clinical record forms or questionnaires. In addition, an umbilical hernia was considered equal to an incisional hernia due to the difficulty to distinguish between umbilical and incisional hernia after RALP.

Questions asked in the clinical record form were: “Has the patient been re-operated due to hernia? (yes/no)” including NOMESCO code (classification of surgical procedures according to Nordic Medico-Statistical committee), and “Has the patient been reoperated for other reason? (yes/no” including NOMESCO code. Patients answered the following questions at intervals described above: “Have you contacted the health care due to groin hernia? (yes/no)”, “Have you contacted the health care due other reasons? (yes/no). If yes—reason for contact”. Have you been re-admitted to hospital? (yes/no). If yes—reason for and date of re-admittance? “Have you had surgery after your prostate surgery during the past year? (yes/no). If yes—reason and date.”

Patients with a present inguinal hernia at the time of prostatectomy reported in the baseline questionnaire were excluded from the analyses as were those who had an inguinal hernia repair at the index prostatectomy, as reported in the perioperative clinical record form.

### Risk factors

Information regarding patient characteristics of importance for inguinal hernia formation was retrieved from the preoperative clinical record form and patient questionnaire, respectively.

Based upon previous studies possibly important variables for developing inguinal hernia such as age, previous inguinal hernia repair, comorbidity in terms of pulmonary disease, diabetes, degree of physical workload, high and low BMI, smoking, level of physical activity, pelvic lymph node dissection and clinical tumor stage [17–20], were retrieved from baseline questionnaire and clinical record forms.

### Statistics

The statistical analyses plan (SAP) defining outcomes, effect measures, possible confounding factors and details about the statistical analyses methods was pre-specified and can be found in Supplementary materials.

The LAPPRO trial was designed with the aim to compare RALP and RRP with regard to urinary leakage at 12 months [16]. Inguinal and incisional hernia formation is a tertiary outcome in that study and the sample size was not calculated with comparison of hernia formation in mind. Sample size was estimated to be 600 in each arm to detect a difference of 30 relative percent with significance level set at. 0.05 and power 80%. Three independent researchers who performed an interim analysis instead suggested unequal group sizes (a 2:1 distribution) of 700 in the radical retropubic prostatectomy group and 1400 in the robotic-assisted laparoscopic prostatectomy group, to be able to detect a difference of five absolute per cent and hence the cohorts differ in sizes.[16].

### Interpretation of results

This is an explorative study, and the results should be interpreted cautiously. The significance level used was 0.05. No adjustment for multiplicity was made.

The modified Poisson regression approach proposed by Zou [21] was used to estimate the relative risk adjusted for different risk factors 24 months after surgery. The results are given as risk ratios for inguinal hernia, two-sided tests with 95% confidence interval and *p* value. Both the unadjusted result, the adjusted result and the model derived by the algorithm by Bursac [22], were calculated and presented.

The Bursac’s method [22] consists of multiple steps and analysis. The first step was to perform bivariate analysis of each risk factor, retaining those with a *p* value < 0.25 for the multivariate analysis. The second step was a multiple analysis retaining variables with a *p* value < 0.10 or confounders (defined as a those which when excluded resulted in change in another variable estimate above 15%). The final step was an iterative procedure where the rejected covariates were refitted and kept if the *p* value < 0.15.

Statistical analyses were performed using SPSS v. 24 and SAS v. 9.4 (SAS Institute Inc., Cary, North Carolina, USA).

## Results

**Fig. 1:**
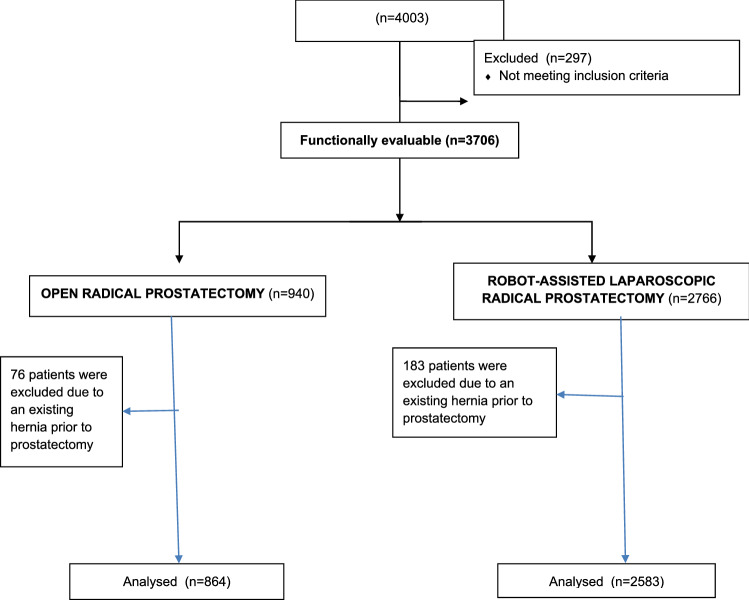
Flowchart LAPPRO Hernia study enrollment: Sept 1st 2008–Nov 7th-2011

Four thousand and three patients were enrolled in the LAPPRO trial between September 2008 and November 2011 out of whom 297 did not meet the inclusion criteria (Fig. 1). For analyses concerning risk of developing inguinal hernia, individuals with a prevailing inguinal hernia at the time of prostatectomy were excluded as were those who had a concomitant inguinal hernia repair at the index prostatectomy, resulting in 3447 evaluable patients.

Correspondingly, four individuals who had an umbilical/incisional hernia repair during prostatectomy were excluded when preparing for analysis of incisional hernia. (flowchart in supplementary material).

Patient characteristics are shown in Table 1. Despite the non-randomised design of the trial, there were no obvious differences between the two groups concerning patient characteristics. Median age in both groups was 63 years and BMI was 26. The majority of patients had a clinical tumor stage characterised as 1 or 2.

**Table 1:** Characteristics of patients in the LAPPRO trial

	Robot-assisted laparoscopic prostatectomy (RALP)	Radical retropubic prostatectomy (RRP)	Total
Patient % (*n*)	2583	864	3447
Age, median years (SD)	63(6.3)	63(5.9)	63(6.2)
Body mass index, median (SD)	25.9 (3.1)	26.2(3.0)	26.0(3.0)
Lymph node dissection			
Yes	337 (13%)	260 (30%)	597 (17%)
No	2238 (87%)	581 (67%)	2819 (82%)
Missing	8 (0%)	23 (3%)	31(0.1%)
Tumor stage			
cT1-2	2452 (95%)	785 (91%)	3237 (94%)
cT3	75 (3%)	33 (4%)	108 (3%)
Missing	56 (2%)	46 (5%)	102(3%)
Diabetes			
Yes	136 (5%)	52 (6%)	188 (5%)
No	2111 (82%)	680 (79%)	2791(81%)
Missing	336 (13%)	132 (15%)	468(14%)
Pulmonary disease			
Yes	52 (2%)	23 (3%)	75 (2%)
No	2190 (85%)	707 (82%)	2897 (84%)
Missing	341 (13%)	134 (16%)	475(14%)
Current smoker			
Yes	228 (9%)	61 (7%)	289 (8%)
No	2022 (78%)	670 (78%)	2692 (78%)
Missing	333 (13%)	133 (15%)	466 (14%)
Previous hernia op			
yes	289 (11%)	98 (11%)	387 (11%)
No	1884 (73%)	607 (70%)	2491 (72%)
Missing	410 (16%)	159 (18%)	569 (17%)
Cardio^1^			
Yes	768 (30%)	252 (29%)	1020 (30%)
No	1479 (57%)	479 (55%)	1958 (57%)
Missing	336 (13%)	133 (4%)	469 (14%)
Physical activity^2^			
Seldom	823 (32%)	449 (52%)	1272(37%)
Often	1433 (55%)	281 (33%)	1714 (50%)
Missing	327 (13%)	134 (16%)	461 (13%)

^1^Indicates patients history or presence of myocardial infarction, angina pectoris, hypertension or heart failure

^2^How often have you been physical active during the last months. Examples are bicycle, walking, gymnastics or equivalent activities (1) never, (2) seldom 1–2/week, (3) often 3–4/week and (4) daily (5–7/week)

Within 24 months, 262 patients developed an inguinal hernia, 189 (7.3%) after robot-assisted laparoscopic prostatectomy and 73 (8.4%) after open radical prostatectomy (Table 2).

**Table 2:** Patients developing inguinal and incisional hernia within 24 months after robot-assisted

	Robot-assisted laparoscopic prostatectomy (RALP)	Open radical retropubic prostatectomy (RRP)	Total
Inguinal hernia after prostatectomy	189/2583 (7.3%)	73/864 (8.4%)	262/3447 (7.6%)
Incisional hernia after prostatectomy	41/2763 (1.5%)	4/939 (0.4%)	45/3702 (1.2%)

Laparoscopic prostatectomy compared and open radical retropubic prostatectomy

The unadjusted estimate resulted in a non-significant 13% (95% CI [0.67; 1.12]) decreased relative risk of developing inguinal hernia within 24 months after robot-assisted laparoscopic radical prostatectomy compared to radical retropubic prostatectomy. Adjusting for the all the identified risk factors or possible cofounders, the risk reduction was 17% (95% CI [0.617; 1.13]), and using the method of Bursac resulted in a 18% (95% CI [0.62; 1.09, non-. significant]) risk reduction (Table 3).

**Table 3:** Multivariate analyses of relative risk of developing an inguinal hernia after prostatectomy

	Full model adjusted analyses			Model adjusted using the Bursac algorithm^2^		
	Relative risk	*p* value	95%CI	Relative risk	*p* value	95% CI
Increasing age	1.03	0.022	0.00–1.05	1.03	0.012	1.01–1.05
Increasing BMI	0.88	< 0001	0.84–0.92	0.88	< 0001	0.84–0.92
Cardiovascular disease	0.76	0.079	0.56–1.03	0.78	0.096	0.57–1.05
Pulmonary disease	1.35	0.407	0.66–2.77			
Diabetes	0.54	0.136	0.24–0.21			
Physical activity	1.02	0.91	0.77–1.35			
Previous inguinal hernia repair	1.37	0.062	0.98–1.92	1.42	0.031	1.03–1.97
Previous abdominal surgery	0.83	0.259	0.59–1.15			
Smoking	0.79	0.353	0.47–1.30			
cT-stage	0.79	0.458	0.43–1.47			
Type of operation	0.83	0.238	0.62–1.13	0.82	0.182	0.62–1.09
Lymph node dissection	1.09	0.64	0.76–1.55			

Adjusted analyses include all variables with a *p* value < 0.25 in univariate analyses and type of operation

Clinically significant risk factors for developing an inguinal hernia repair were higher age, lower BMI and a previous inguinal hernia repair.

Incisional hernia was reported in four (0.4%) patients after open radical retropubic prostatectomy and 41 (1.5%) after robot-assisted laparoscopic prostatectomy (Table 2). Due to the low number of observations, no statistical analysis was made.

## Discussion

We found no significant difference in inguinal hernia formation after open and robot-assisted laparoscopic radical prostatectomy. Regarding incisional hernia, the incidence was low in both groups.

The incidence of inguinal hernia within 24 months assessed in our study (7.3% and 8.4%) after prostatectomy was lower than reported in earlier studies, where postoperative incidences of inguinal hernia varied between 12–25% [1, 8, 23]. Others have suggested incidences in line with our results [24, 25], but the reported incidence rates have varied greatly between studies ever since the condition was first reported by Reagan et al. [1]. The reported incidence rates demonstrate the heterogeneity between the methods of detection of both prevalent inguinal hernias at the time of surgery and postoperatively occurring hernias. In a review article from 2003, Higgins et al. concluded that the synthesized 2-year incidence rate from eight studies was 11.1%, with a 95% confidence interval of 8.2–14.0% and a *I*^2^ = 92.9% in a *Q*-test of heterogeneity [26]. In our study we asked the patients preoperatively about inguinal hernia and for analyses excluded all patients with a prevalent hernia at the time of prostatectomy, in total 7% (251/3706). In prior studies with a cross-sectional design information regarding clinical or sub-clinical inguinal hernias at the time of prostate surgery is often lacking [5, 8]. Pre-existing hernias could then be found at follow-up, and be included in the postoperative incidence. This could in part explain why our incidence is in the lower range of the previously published figures. Furthermore, some studies had a longer follow-up period than two years. Two Swedish nationwide register studies had a follow-up of six years and in both, the incidence continued to increase after 2 years follow-up [5, 8] up to 11% after 6 years [5]. Compared to the control group, consisting of healthy men, the risk of being operated due to inguinal hernia was tripled [5]. The risk of developing inguinal hernia after prostatectomy was high in both groups but significantly higher after open radical retropubic prostatectomy compared to minimally invasive procedure [5].

We found no difference in the incidence of inguinal hernia formation postoperatively comparing the open to the robot-assisted laparoscopic group. The difference in outcome, inguinal hernia operation rather than hernia development, and shorter follow-up in this study could be explanations. However, in a recent nationwide study both diagnosis and hernia operation were accounted for with no significant difference between open and robot-assisted laparoscopic operation [8].

A low BMI, high age and previous hernia repair was found to be associated with an increased risk of inguinal hernia formation as have others [18, 19, 27].

In our study there were few observations of incisional hernias (0.4% and 1.5% after RRP and RALP, respectively), in line with previous studies. However in two earlier studies the cohort sizes were large enough to find robot-assisted technique to be associated with a statistically significant increased risk of incisional hernia formation [8, 11]. We would argue that incidence levels as low as that would be of very limited clinical significance and that statistics therefore is of no use.

Strengths of the study was the cohort size, the prospective and controlled design as well as the pre- and postoperative patient reports including data on pre-existing comorbidity such as inguinal and incisional hernia. The high response rates on questionnaires and clinical record forms were a clear strength [28, 29]. Information regarding inguinal and incisional hernia were retrieved both from reports by the surgeon and the patient at each point of follow-up.

The non-randomised design was a weakness, however, counteracted by the extensive data collection in combination with a high response rate which made it possible to adjust for possible differences between the groups at analyses. Questions regarding groin hernia were included in patient questionnaires and clinical record forms from the start of the study. However, hernia was not the primary endpoint of the LAPPRO trial which is a limitation when assessing incisional hernia formation after prostatectomy. Likewise the power of the study was calculated with respect to urinary leakage with 2:1 distribution for RALP and RRP resulting in uneven cohorts potentially reducing the power of the study to detect hernia.

## Conclusion

This study could not confirm previous reports of differences in inguinal hernia formation after open and robot-assisted radical prostatectomy, respectively. The cohort size in combination with the detailed data collection suggests that any difference is likely to be negligible and the study can constitute an important addition in upcoming meta-analyses. Until then risk of hernia formation cannot be a reason for choosing either surgical technique.

## Electronic supplementary material

Below is the link to the electronic supplementary material.

Supplementary material 1 (PDF 286 kb)

Supplementary material 2 (PDF 784 kb)

## References

[1] Regan TC, Mordkin RM, Constantinople NL, Spence IJ, Dejter SW (1996). Incidence of inguinal hernias following radical retropubic prostatectomy. Urology.

[2] Lodding P, Bergdahl C, Nyberg M, Pileblad E, Stranne J, Hugosson J (2001). Inguinal hernia after radical retropubic prostatectomy for prostate cancer: a study of incidence and risk factors in comparison to no operation and lymphadenectomy. J Urol.

[3] Stranne J, Lodding P (2011). Inguinal hernia after radical retropubic prostatectomy: risk factors and prevention. Nat Rev Urol.

[4] Zhu S, Zhang H, Xie L, Chen J, Niu Y (2013). Risk factors and prevention of inguinal hernia after radical prostatectomy: a systematic review and meta-analysis. J Urol.

[5] Nilsson H, Stranne J, Stattin P, Nordin P (2013). Incidence of groin hernia repair after radical prostatectomy: a population-based nationwide study. Ann Surg.

[6] Stranne J, Hugosson J, Lodding P (2007). Inguinal hernia is a common complication in lower midline incision surgery. Hernia.

[7] Stranne J, Johansson E, Nilsson A, Bill-Axelson A, Carlsson S, Holmberg L (2010). Inguinal hernia after radical prostatectomy for prostate cancer: results from a randomized setting and a nonrandomized setting. Eur Urol.

[8] Fridriksson JO, Folkvaljon Y, Lundstrom KJ, Robinson D, Carlsson S, Stattin P (2017). Long-term adverse effects after retropubic and robot-assisted radical prostatectomy. Nationwide, population-based study. J Surg Oncol..

[9] Ku JY, Lee CH, Park WY, Lee NK, Baek SH, Ha HK (2018). The cumulative incidence and risk factors of postoperative inguinal hernia in patients undergoing radical prostatectomy. Int J Clin Oncol.

[10] Carlsson SV, Ehdaie B, Atoria CL, Elkin EB, Eastham JA (2013). Risk of incisional hernia after minimally invasive and open radical prostatectomy. J Urol.

[11] Hermann M, Gustafsson O, Sandblom G (2017). Incidence of incisional hernia after minimally invasive and open radical prostatectomy: a population-based nationwide study. Scand J Urol.

[12] van't Riet M, Steyerberg EW, Nellensteyn J, Bonjer HJ, Jeekel J (2002). Meta-analysis of techniques for closure of midline abdominal incisions. Br J Surg.

[13] Brooks NA, Boland RS, Strigenz ME, Mott SL, Brown JA (2018). Nongenitourinary complications associated with robot-assisted laparoscopic and radical retropubic prostatectomy: a single institution assessment of 1,100 patients over 11 years. Urol Oncol.

[14] Nilsson H, Stylianidis G, Haapamaki M, Nilsson E, Nordin P (2007). Mortality after groin hernia surgery. Ann Surg.

[15] Nilsson H, Angeras U, Sandblom G, Nordin P (2016). Serious adverse events within 30 days of groin hernia surgery. Hernia.

[16] Thorsteinsdottir T, Stranne J, Carlsson S, Anderberg B, Bjorholt I, Damber JE (2011). LAPPRO: a prospective multicentre comparative study of robot-assisted laparoscopic and retropubic radical prostatectomy for prostate cancer. Scand J Urol Nephrol.

[17] Read RC (1998). Cigarette smoking, herniation, and recurrence. Surgery.

[18] Hemberg A, Holmberg H, Norberg M, Nordin P (2017). Tobacco use is not associated with groin hernia repair, a population-based study. Hernia.

[19] Rosemar A, Angeras U, Rosengren A, Nordin P (2010). Effect of body mass index on groin hernia surgery. Ann Surg.

[20] Zendejas B, Hernandez-Irizarry R, Ramirez T, Lohse CM, Grossardt BR, Farley DR (2014). Relationship between body mass index and the incidence of inguinal hernia repairs: a population-based study in Olmsted County, MN. Hernia.

[21] Zou G (2004). A modified poisson regression approach to prospective studies with binary data. Am J Epidemiol.

[22] Bursac Z, Gauss CH, Williams DK, Hosmer DW (2008). Purposeful selection of variables in logistic regression. Source Code Biol Med.

[23] Ichioka K, Yoshimura K, Utsunomiya N, Ueda N, Matsui Y, Terai A (2004). High incidence of inguinal hernia after radical retropubic prostatectomy. Urology.

[24] Yoshimine S, Miyajima A, Nakagawa K, Ide H, Kikuchi E, Oya M (2010). Extraperitoneal approach induces postoperative inguinal hernia compared with transperitoneal approach after laparoscopic radical prostatectomy. Jpn J Clin Oncol.

[25] Lepor H, Robbins D (2007). Inguinal hernias in men undergoing open radical retropubic prostatectomy. Urology.

[26] Higgins JP, Thompson SG, Deeks JJ, Altman DG (2003). Measuring inconsistency in meta-analyses. BMJ.

[27] Primatesta P, Goldacre MJ (1996). Inguinal hernia repair: incidence of elective and emergency surgery, readmission and mortality. Int J Epidemiol.

[28] Haglind E, Carlsson S, Stranne J, Wallerstedt A, Wilderang U, Thorsteinsdottir T (2015). Urinary incontinence and erectile dysfunction after robotic versus open radical prostatectomy: a prospective, controlled nonrandomised trial. Eur Urol.

[29] Sooriakumaran P, Pini G, Nyberg T, Derogar M, Carlsson S, Stranne J (2018). Erectile function and oncologic outcomes following open retropubic and robot-assisted radical prostatectomy: results from the laparoscopic prostatectomy robot open trial. Eur Urol.

