# Correction: Risk of hernia formation after radical prostatectomy: a comparison between open and robot-assisted laparoscopic radical prostatectomy within the prospectively controlled LAPPRO trial

**DOI:** 10.1007/s10029-022-02593-y

**Published:** 2022-03-11

**Authors:** H. Nilsson, J. Stranne, J. Hugosson, C. Wessman, G. Steineck, A. Bjartell, S. Carlsson, T. Thorsteinsdottir, S. I. Tyritzis, A. Lantz, P. Wiklund, E. Haglind

**Affiliations:** 1 Department of Surgery, Institute of Clinical Sciences, SSORG, Scandinavian Surgical Outcomes Research Group, Sahlgrenska Academy, University of Gothenburg, Gothenburg, Sweden; 2 Department of Surgery, Region Västra Götaland, Sahlgrenska University Hospital, Gothenburg, Sweden; 3 Department of Urology, Institute of Clinical Science, Sahlgrenska Academy, University of Gothenburg, Gothenburg, Sweden; 4 Department of Urology, Region Västra Götaland, Sahlgrenska University Hospital, Gothenburg, Sweden; 5 Sweden and Health Metrics Unit, The Sahlgrenska Academy, University of Gothenburg, Gothenburg, Sweden; 6 Division of Clinical Cancer Epidemiology Institute of Clinical Sciences, Sahlgrenska Academy At the University of Gothenburg, Gothenburg, Sweden; 7 Department of Oncology and Pathology, Division of Clinical Cancer Epidemiology, Karolinska Institutet, Gothenburg, Sweden; 8 Department of Urology, Skåne University Hospital, Lund University, Gothenburg, Sweden; 9 Faculty of Nursing, Research Institute in Emergency Care, Landspitali University Hospital, University of Iceland, Reykjavik, Iceland; 10 Department of Molecular Medicine and Surgery, Section of Urology, Karolinska Institutet, Gothenburg, Sweden; 11 Department of Surgery, Sahlgrenska University Hospital/Östra, Östra Sjukhuset, 416 85 Göteborg, Sweden

## Correction to: Hernia (2020) 26:157–164 10.1007/s10029-020-02178-7

The originally published article without open choice elements. Now it has been corrected.

The original article has been corrected.

