# Supplementary figures and images for: Risk of hernia formation after radical prostatectomy: a comparison between open and robot-assisted laparoscopic radical prostatectomy within the prospectively controlled LAPPRO trial
Source: Hernia. 2020 Apr 11;26(1):157–64. doi: 10.1007/s10029-020-02178-7 (PMC8881255; doi:10.1007/s10029-020-02178-7)

## Flow Chart

### LAPPRO Incisional Hernia Study

#### Enrollment

Start Date: September 1, 2008

End Date: November 7, 2011

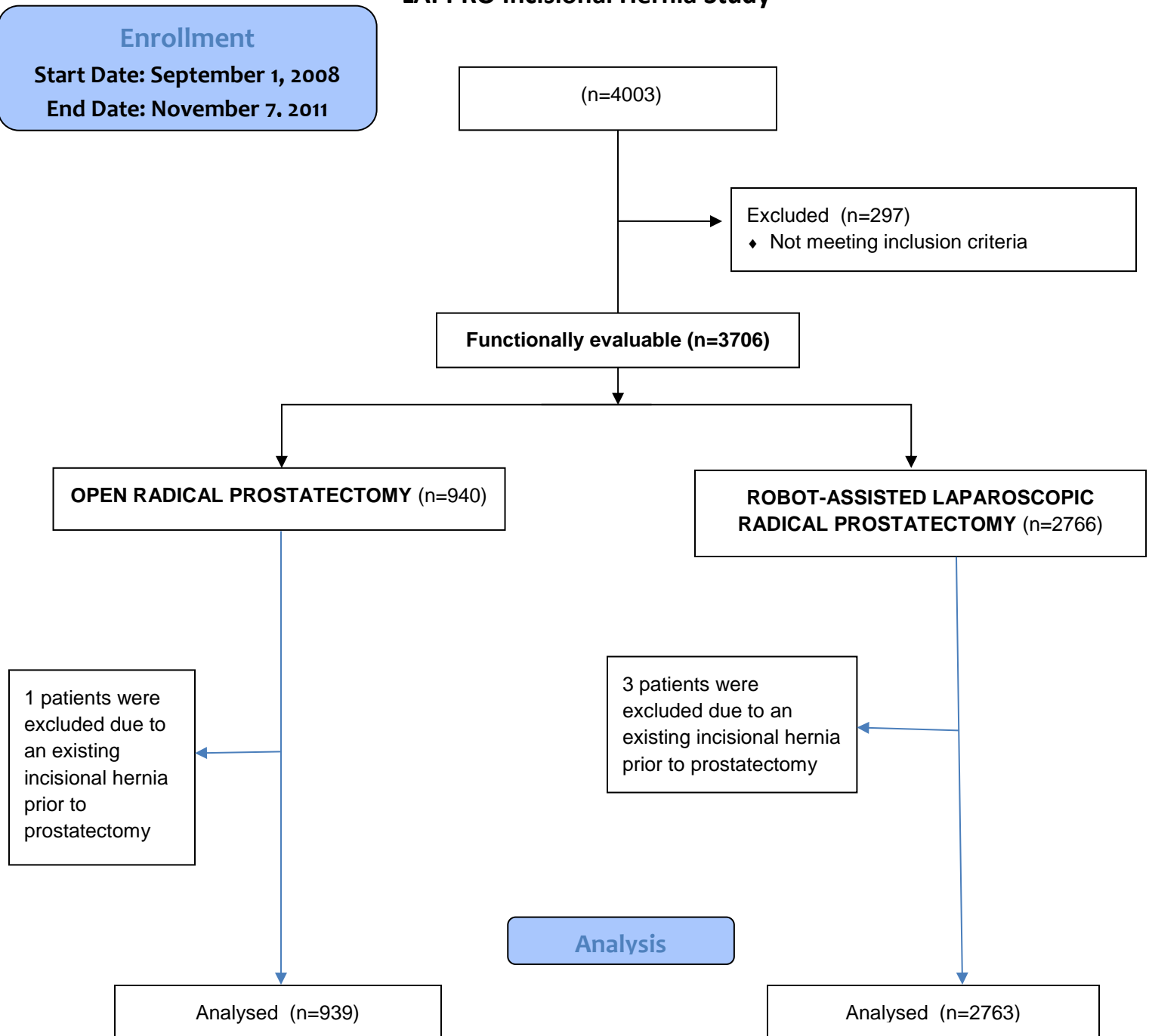

Supplement: Supplementary file 1 — Supplementary material 1 (PDF 286 kb) [file 10029_2020_2178_MOESM1_ESM.pdf]
