# Supplementary material for: Risk of hernia formation after radical prostatectomy: a comparison between open and robot-assisted laparoscopic radical prostatectomy within the prospectively controlled LAPPRO trial
Source: Hernia. 2020 Apr 11;26(1):157–64. doi: 10.1007/s10029-020-02178-7 (PMC8881255; doi:10.1007/s10029-020-02178-7)
Supplement: Supplementary file 2 — Supplementary material 2 (PDF 784 kb) [file 10029_2020_2178_MOESM2_ESM.pdf]

Scandinavian Surgical Outcomes Research Group
www.ssorg.net

Date

Statistical Analysis Plan

Risk of developing an inguinal and incisional hernia after radical prostatectomy. Results in a prospective controlled trial (LAPPRO)

VERSION 1.2

Nilsson H, Bjartell A, Carlsson S, Hugosson J, Steineck G, Thorsteinsdottir T, Tyrizis S, Stranne J,Wallerstedt A, Wessman C, Wiklund P, Eva Haglind

Table of contents

Statistical Analysis Plan 1
INTRODUCTION 2
ANALYSIS OBJECTIVES 2
STUDY METHODS 2
ANALYSIS POPULATION, TYPE OF ANALYSIS 3
Analysis population 3
VARIABLES AND ENDPOINTS 3
Primary objective 3
Covariates, stratum and subgroups 7
HANDLING OF MISSING VALUES AND OTHER DATA CONVENTIONS 7
STATISTICAL METHODOLOGY 8
General principles 8
Multiplicity 8
Interpretation of results 8
Sample size 8
Demography and patient characteristics 8
Statistical method 8
LIST OF TABLES AND FIGURES PLANNED FOR THE MANUSCRIPT 9
REFERENCES 9

INTRODUCTION

The LAPPRO trial, a prospective, controlled non-randomized trial where robot assisted laparoscopic prostatectomy was compared with open retropubic prostatectomy, is the context for the described study, a secondary outcome of the trial. Inclusion took place during September 2008 until November 2011, with a total of 4003 included patients at 14 Swedish Departments of urology.

Since the middle of 1990s several studies have reported an increased risk for developing an inguinal hernia after prostatectomy [1-6] compared with the normal population. Patients operated with open radical retropubic prostatectomy (RRP) or robot assisted prostatectomy (RALP), had a threefold increased risk of being operated because of inguinal hernia [7]. Hence, evidence suggests an increased incidence of inguinal hernia even after minimal invasive surgery such as RALP[8]. But as some studies suggest the risk to be less with RALP compared to RRP[7] this study of a very large cohort of men operated by either retropubic or robot assisted laparoscopic prostatectomy will shed further light on this question.

The primary aim of this study is to assess the difference in risk of developing an inguinal hernia within 2 years of a prostatectomy comparing the techniques of RRP and RALP.

This statistical analysis plan was completed and finalized before the personnel involved in the project started the analyses of data.

ANALYSIS OBJECTIVES

The primary objective is to compare the risk of developing an inguinal hernia after open RRP respectively RALP within 2 years.

The secondary objective is to evaluate potential risk factors for developing an inguinal hernia, after prostatectomy

STUDY METHODS

We will use data from the entire cohort in the LAPPRO trial (n=3706). Baseline characteristic of patients and operation is retrieved through perioperative CRF and questionnaire. Information regarding postoperative formation of an inguinal hernia is retrieved through patient questionnaires 6-12 weeks, 12 months and 24 months after surgery as well as CRF answered by doctor at clinical follow-up 12 weeks, 12 months and 24 months after surgery.

Specific questions to be analysed are listed in the section of Variables and Endpoints.

ANALYSIS POPULATION, TYPE OF ANALYSIS

Analysis population

The entire cohort in the LAPPRO trial (n=3706), where RALP was compared with RRP. Since it is a non-randomized study patient will be analysed as-treated.

VARIABLES AND ENDPOINTS

Primary objective

The estimation of the incidence of hernia formation after prostatectomy is based on physician reports of surgery due to inguinal hernia and patient reports on seeking health care due to suspected inguinal hernia.

PRE/PERIOPERATIVE INDICATION OF GROIN HERNIA IS BASED UPON:

Preoperative questionnaire

If the patient has, or have had, an inguinal hernia (QoL1, Q41 and Q46).

CRF perioperatively
Item 18-23: Other, same time surgery,

PHYSICIAN REPORTS OF SURGERY DUE TO INGUINAL HERNIA ARE BASED ON:

CRF perioperatively
Item 18-23: Other, same time surgery,
CRF 6-12 weeks
Item 47-48: Has the patient been re-operated due to hernia? (Yes/No). Date and NOMESCO code JAB 10, 11, 20, 30, 40, 96, 97
Item 49-50: Has the patient been reoperated for other reason? (Yes/No). Date and NOMESCO code JAB 10, 11, 20, 30, 40, 96, 97
CRF 12 months
Item 38-40: Has the patient been re-operated due to inguinal hernia? (Yes/No). Date and NOMESCO code JAB 10, 11, 20, 30, 40, 96, 97
Item 41-49: Has the patient been reoperated for other reason? (Yes/No). Date and NOMESCO code JAB 10, 11, 20, 30, 40, 96, 97
CRF 24 months
Item 38-40: Has the patient been re-operated due to inguinal hernia? (Yes/No). Date and NOMESCO code JAB 10, 11, 20, 30, 40, 96, 97
Item 41-49: Has the patient been reoperated for other reason? (Yes/No). Date and NOMESCO code JAB 10, 11, 20, 30, 40, 96, 97

PATIENT REPORTS ON SEEKING HEALTH CARE DUE TO SUSPECTED INGUINAL HERNIA ARE BASED ON THE FOLLOWING:

Questionnaire three months:
Q 22: Have you contacted the health care due to groin hernia? (Yes/No)
Q 31: Have you contacted the health care due other reasons? (Yes/No). If yes - reason for contact.
Q 46-54: Have you been re-admitted to hospital? (Yes/No). If yes – reason for and date of re-admittance? Only applicable if date is after prostate surgery date.

Questionnaire 12 months
Q 39: Have you contacted the health care due to groin hernia? (Yes/No)
Q 47: Have you contacted the health care due other reasons? (Yes/No). If yes - reason for contact.
Q 48-66: Have you been re-admitted to hospital? (Yes/No). If yes – reason for and date of re-admittance? Only applicable if date is after prostate surgery date.
Q 67-79: Have you had surgery after your prostate surgery during the past year? (Yes/No). If yes – reason and date.

Questionnaire 24 months
Q 50: Have you contacted the health care due to groin hernia? (Yes/No)
Q 58: Have you contacted the health care due other reasons? (Yes/No). If yes - reason for contact.
Q 59-77: Have you been re-admitted to hospital? (Yes/No). If yes – reason for and date of re-admittance? Only applicable if date is after prostate surgery date.
Q 78-90: Have you had surgery after your prostate surgery during the past year? (Yes/No). If yes – reason and date.

If at least one of the CRF reports indicates the patient has been re-operated or at least one of the items in the questionnaire indicates a suspected hernia formation, then the outcome will be defined as the occurrence of hernia (positive outcome).

If neither the CRF nor the questionnaire reported events related to inguinal hernia formation, then the outcome will be defined as the no occurrence of hernia (negative outcome).

Missing values will be interpreted as absence of hernia

The primary endpoint is proportion of patients with at least one inguinal hernia within 24 months after surgery.

Possible preoperative risk factors for inguinal herniaThe following risk factors for developing an inguinal hernia will be evaluated:

1. Age
2. Physical workload
3. BMI
4. Diabetes
5. Pulmonal disease
6. Smoking/no smoking
7. Hernia occurrence/operation prior to prostatectomy.
8. Physical activity

9. Preoperative tumour risk category defined as follows:
   Low risk T1-2 vs high risk T3-4

BASELINE PATIENT CHARACTERISTICS, TUMOUR FACTORS, COMORBIDITIES AND RISK FACTORS ARE BASED UPON:

Preoperative questionnaire

Sociodemografics (Q4-6), BMI (Q8-9), relevant comorbidities (Q30, 35, 41, 43, 44), previous abdominal surgery (Q52), previous IH surgery (Q46, 47), smoking (Q57), physical activity (Q143), urinary flow (Q149)

Patients were dichotomized as current smoker (including pipe smoking) or non-smoker (former smoker or never smoker) according to their answer to the question "*Do you smoke or have you smoked?*".

Q 143: Hur ofta har Du varit fysiskt aktivt i 30 minuter eller mer den senaste månaden..? *With a 4 point Likert scale (1= aldrig, 2=ibland (1-2 ggr/v), 3=Ofta (3-4 ggr/v), 4= Dagligen eller nästan dagligen (5-7 ggr/vecka)) The answers will be dichotomized with a cut-off point between 2 and 3 prior to analysis.*
Q 146: Hur ofta har Du varit varit tvungen att trycka på eller eller krysta för att börja kissa den senaste månaden? *With a 6 point Likert scale (1=aldrig, 2=färre än 1 av 5 vattenkastningar, 3= färre än hälften av vattenkastningar, 4= ungefär hälften av vattenkastningar, 5= fler än hälften av vattenkastningar, 6=alltid). The answers will be dichotomized with a cut-off point between 3 and 4 prior to analysis.*

Questionnaire three months:
Q 8: Har ditt arbete varit kroppsligt aktivt den senaste månaden? *With a 6 point Likert scale (0=Inte aktuellt jag jobbar inte, 1= nej inte kroppsligt aktivt alls, 2=ja, lite kroppsligt aktivt, 3=Ja måttligt kroppsligt aktivt, 4= Ja, mycket kroppsligt aktivt, 5= annat) The answers will be dichotomized with a cut-off point between 2 and 3 prior to analysis.*
Q 205: Hur ofta har Du varit fysiskt aktivt i 30 minuter eller mer den senaste månaden..? *With a 4 point Likert scale (1= aldrig, 2=ibland (1-2 ggr/v), 3=Ofta (3-4 ggr/v), 4= Dagligen eller nästan dagligen (5-7 ggr/vecka)) The answers will be dichotomized with a cut-off point between 2 and 3 prior to analysis.*
Q 221: Hur ofta har Du varit varit tvungen att trycka på eller eller krysta för att börja kissa den senaste månaden? *With a 6 point Likert scale (1=aldrig, 2=färre än 1 av 5 vattenkastningar, 3= färre än hälften av vattenkastningar, 4= ungefär hälften av vattenkastningar, 5= fler än hälften av vattenkastningar, 6=alltid). The answers will be dichotomized with a cut-off point between 3 and 4 prior to analysis.*

Questionnaire 12 months
Q 12: Har ditt arbete varit kroppsligt aktivt den senaste månaden? *With a 6 point Likert scale (0=Inte aktuellt jag jobbar inte, 1= nej inte kroppsligt aktivt alls, 2=ja, lite kroppsligt aktivt, 3=Ja*

måttligt kroppsligt aktivt, 4= Ja, mycket kroppsligt aktivt, 5= annat) The answers will be dichotomized with a cut-off point between 2 and 3 prior to analysis.
Q 219: Hur ofta har Du varit fysiskt aktivt i 30 minuter eller mer den senaste månaden..? *With a 4 point Likert scale (1= aldrig, 2=ibland (1-2 ggr/v), 3=Ofta (3-4 ggr/v), 4= Dagligen eller nästan dagligen (5-7 ggr/vecka)) The answers will be dichotomized with a cut-off point between 2 and 3 prior to analysis.*
Q 235: Hur ofta har Du varit varit tvungen att trycka på eller eller krysta för att börja kissa den senaste månaden? *with a 6 point Likert scale (1=aldrig, 2=färre än 1 av 5 vattenkastningar, 3= färre än hälften av vattenkastningar, 4= ungefär hälften av vattenkastningar, 5= fler än hälften av vattenkastningar, 6=alltid). The answers will be dichotomized with a cut-off point between 3 and 4 prior to analysis. Interpreted as risk factor and included in analyses if answered "yes" on same or previous questionnaire as inguinal hernia is identified.*

Questionnaire 24 months
Q 23: Har ditt arbete varit kroppsligt aktivt den senaste månaden? *With a 6 point Likert scale (0=Inte aktuellt jag jobbar inte, 1= nej inte kroppsligt aktivt alls, 2=ja, lite kroppsligt aktivt, 3=Ja måttligt kroppsligt aktivt, 4= Ja, mycket kroppsligt aktivt, 5= annat) The answers will be dichotomized with a cut-off point between 2 and 3 prior to analysis.*
Q 230: Hur ofta har Du varit fysiskt aktivt i 30 minuter eller mer den senaste månaden..? *With a 4 point Likert scale (1= aldrig, 2=ibland (1-2 ggr/v), 3=Ofta (3-4 ggr/v), 4= Dagligen eller nästan dagligen (5-7 ggr/vecka)) The answers will be dichotomized with a cut-off point between 2 and 3 prior to analysis.*
Q 256: Hur ofta har Du varit varit tvungen att trycka på eller eller krysta för att börja kissa den senaste månaden? *With a 6 point Likert scale (1=aldrig, 2=färre än 1 av 5 vattenkastningar, 3= färre än hälften av vattenkastningar, 4= ungefär hälften av vattenkastningar, 5= fler än hälften av vattenkastningar, 6=alltid). The answers will be dichotomized with a cut-off point between 3 and 4 prior to analysis. Interpreted as risk factor if answered "yes" on same or previous questionnaire as inguinal hernia is identified. Interpreted as risk factor and included in analyses if answered "yes" on same or previous questionnaire as inguinal hernia is identified*

CRF preoperatively
Item 6, clinical stage (cT1-T4)
Item 8, ASA-classification
Item 9, PSA
Item 20, biopsy Gleason score
CRF perioperatively
Item 1-2, Age at surgery
Item 17 Type of surgery
Item 18-23 Other procedures during prostate surgery

Covariates, stratum and subgroups

No additional variables apart from the variables that will be evaluated for risk factors will be included as covariates in the statistical model.

Key Secondary objective

A second object is to analyse the primary variable with the same possible risk factors as above, but include Lymph node dissection in the model.

Item 31-33 Lymph node dissection:,yes vs no

CRF 6-12 weeks
Item 51: Patient drop-out (Yes/No)
CRF 12 months
Item 51: Patient drop-out (Yes/No)
CRF 24 months
Item 51: Patient drop-out (Yes/No)

Secondary objectives

The estimation of the incidence of incisional hernia formation after prostatectomy is based on physician reports of surgery due to incisional hernia and patient reports on seeking health care due to suspected incisional hernia.

Possible risk factors for incisional hernia ?

Age above 70
Artherosclerosis
BMI above 30
Hypertension
Previous abdominal surgery
Smoking
Diabetes
Pulmonary disease

HANDLING OF MISSING VALUES AND OTHER DATA CONVENTIONS

In order to handle the issue with missing data of risk factors used in the model, multiple imputation by means of chained equations (MICE) will be used.

STATISTICAL METHODOLOGY

General principles

Multiplicity

Interpretation of results

A risk factor is here referred to as a variable that is associated with the outcome of interest. The variables specified in the previous sections are, based on clinical judgement and earlier studies, regarded as potentially clinically relevant. Interpretation of the results will consider clinical relevance of the estimated degree of association and will be cautiously addressed due to the risk of spurious and false positive associations.

Sample size

The LAPPRO study was designed with the aim to compare RALP and RRP with regard to incontinence at 12 months [9]. Groin hernia formation is a secondary outcome in that study and hence sample size is not calculated for groin hernia formation.

In a previous study[7] the incidence of inguinal hernia operation within two years after prostatectomy was approximately 6% and 7.5% for RALP and RRP (a risk ratio of 0.80), respectively. With 3706 evaluable patients (2764 and 942 with RALP and RRP, respectively) and a true incidence of 6% and 7.5%, these will be detected with approximately 35% power. However, for the endpoint used here, the incidence is expected to be higher. As illustration, with true incidences of 10% and 12.5%, respectively, a difference will be detected with 55% power using a two-sided test at 5% significance level.

Demography and patient characteristics

The following variables will be summarized and described:

- Age (median (Q1 ; Q3))
- BMI (median (Q1 ; Q3))
- Diabetes yes/no
- Cardiopulmonary disease Yes/no
- Smoking/non-smoking
- Heavy work load yes /no
- Tumour stage: low risk, and high risk
- Physical activity in history

Statistical method

The primary objective of evaluating the association between hernia incidence and operation and the evaluation of risk factor will be done simultaneously with the same statistical model. The relative risk of at least one inguinal hernia within 24 months after surgery, comparing surgical methods (RALP, RRP), will be modelled using a modified Poisson regression approach of Zou ([10]).

Except for the exposure, the surgical method (RALP, RRP), possible risk factors (age, physical workload, BMI, diabetes, pulmonal disease, smoking, hernia prior operation, physical activity and tumour size) will be evaluated by the same algorithm introduced by Bursac ("Purposeful selection of variables in logistic regression", Source Code for Biology and Medicine20083:17). This model might also include interactions.

The key secondary objective will evaluate the same risk factors, but will include Lymph node dissection (yes/no) in the model.

The secondary objectives will be evaluated in an analogous way, using the risk factors …. .

LIST OF TABLES AND FIGURES PLANNED FOR THE MANUSCRIPT

Figure 1: Flow chart of included patients

Table 1: Patient characteristics and demography.

Table 2 Multivariate analyses

REFERENCES

1. Sun, M., et al., *Comparative study of inguinal hernia repair after radical prostatectomy, prostate biopsy, transurethral resection of the prostate or pelvic lymph node dissection.* J Urol, 2010. **183**(3): p. 970-5.
2. Lughezzani, G., et al., *Comparative study of inguinal hernia repair rates after radical prostatectomy or external beam radiotherapy.* Int J Radiat Oncol Biol Phys, 2010. **78**(5): p. 1307-13.
3. Ichioka, K., et al., *High incidence of inguinal hernia after radical retropubic prostatectomy.* Urology, 2004. **63**(2): p. 278-81.
4. Stranne, J., et al., *Inguinal hernia after radical prostatectomy for prostate cancer: results from a randomized setting and a nonrandomized setting.* Eur Urol, 2010. **58**(5): p. 719-26.
5. Stranne, J., et al., *Inguinal hernia in stage M0 prostate cancer: a comparison of incidence in men treated with and without radical retropubic prostatectomy--an analysis of 1105 patients.* Urology, 2005. **65**(5): p. 847-51.
6. Lodding, P., et al., *Inguinal hernia after radical retropubic prostatectomy for prostate cancer: a study of incidence and risk factors in comparison to no operation and lymphadenectomy.* J Urol, 2001. **166**(3): p. 964-7.

7. Nilsson, H., et al., *Incidence of Groin Hernia Repair After Radical Prostatectomy: A Population-Based Nationwide Study.* Ann Surg, 2013.
8. Shimbo, M., et al., *Incidence, Risk Factors and a Novel Prevention Technique for Inguinal Hernia after Robot-Assisted Radical Prostatectomy.* Urol Int, 2017. **98**(1): p. 54-60.
9. Thorsteinsdottir, T., et al., *LAPPRO: a prospective multicentre comparative study of robot-assisted laparoscopic and retropubic radical prostatectomy for prostate cancer.* Scand J Urol Nephrol, 2011. **45**(2): p. 102-12.
10. Bursac (“Purposeful selection of variables in logistic regression”, Source Code for Biology and Medicine20083:17
